# Loss of DUSP4 Expression as a Prognostic Biomarker in Clear Cell Renal Cell Carcinoma

**DOI:** 10.3390/diagnostics11101939

**Published:** 2021-10-19

**Authors:** Seongsik Bang, Seungyun Jee, Hwangkyu Son, Young Chan Wi, Hyunsung Kim, Hosub Park, Jaekyung Myung, Su-Jin Shin, Seung Sam Paik

**Affiliations:** 1 Department of Pathology, Hanyang University Seoul Hospital, Hanyang University College of Medicine, Seoul 04763, Korea; grypony@naver.com (S.B.); Jee.seung.yun@gmail.com (S.J.); ganzi4900@gmail.com (H.S.); hhnt5841@gmail.com (H.K.); Parkhstm@gmail.com (H.P.); tontos016@naver.com (J.M.); 2 Department of Pathology, Eunpyeong St. Mary’s Hospital, The Catholic University of Korea, Seoul 03312, Korea; wiyoungchan@gmail.com; 3 Department of Pathology, Gangnam Severance Hospital, Yonsei University College of Medicine, Seoul 06273, Korea; CHARM@yuhs.ac

**Keywords:** renal cell carcinoma, DUSP4, dual-specificity protein phosphatase 4, prognosis

## Abstract

Dual-specificity protein phosphatase 4 (DUSP4) is a negative regulator of mitogen-activated protein kinases. The prognostic impact of DUSP4 expression in renal cell carcinoma is not well studied. Therefore, we evaluated the clinicopathological implications of DUSP4 expression in clear cell renal cell carcinoma by performing immunohistochemistry (IHC). The clinical outcome according to DUSP4 expression was evaluated through survival analyses, and the association between mRNA expression and prognosis was confirmed by online analysis (Kaplan–Meier plotter). Loss of DUSP4 expression was noted in most histological subtypes of renal cell carcinoma. Loss of DUSP4 expression in clear cell renal cell carcinoma was significantly correlated with old age (*p* = 0.033), high histologic grade (*p* < 0.001), tumor necrosis (*p* < 0.001), and high pT category (*p* < 0.001). In survival analysis, loss of DUSP4 expression was associated with poor clinical outcomes in cancer-specific survival and recurrence-free survival (*p* = 0.010 and *p* = 0.007, respectively). Upon TCGA data analysis, patients with low DUSP4 mRNA expression showed a shorter overall survival (*p* = 0.023). These results suggest that loss of DUSP4 expression can be used as a potential biomarker for predicting clinical outcomes in clear cell renal cell carcinoma patients.

## 1. Introduction

Renal cell carcinoma (RCC) originates from the renal tubular epithelium and accounts for the majority of primary kidney cancers. According to global databases, RCC contributes to approximately 2% of cancer diagnoses and deaths, and the incidence is increasing rapidly in developed countries [1]. In the pathogenesis of RCC, a dysregulated metabolic pathway is involved, and genetic alterations such as loss of von Hippel Lindau (VHL) gene function were found in clear cell renal cell carcinoma (ccRCC), the most common type among renal cell carcinomas [2]. Various studies on promising biomarkers were established based on the understanding of molecular pathways of RCC, and carbonic anhydrase IX and VEGF are recognized as prognostic markers or therapeutic targets [3,4]. Additionally, the association between novel biomarkers (CXCL16, ADAM10, B7-H1, Ki-67, survivin, P53, GLUT-1, calveolin-1, and endoglin) and the clinical outcome of RCC patients is being validated [5].

Dual-specificity protein phosphatase 4 (DUSP4) is a negative regulator that dephosphorylates and inactivates mitogen-activated protein kinases (MAPKs) [6]. DUSP4 is located mainly in the nucleus and selectively acts on extracellular-signal-regulated kinase (ERK) and c-Jun N-terminal kinase (JNK) [7,8]. The MAPK family affects cell responses to signals and is involved in various cellular processes such as cell proliferation, survival, and migration [9]. Therefore, many studies on dysregulated DUSP4 expression and cancer have been performed. In several types of cancer, DUSP4 expression affects carcinogenesis and drug resistance; thus, DUSP4 is considered a candidate prognostic marker or therapeutic target [10,11,12,13]. However, its exact molecular mechanism and clinicopathologic role in cancer are unknown.

Recently, Zeng et al. reported that mRNA level of DUSP4 was increased in ccRCC tissue and cell lines. They showed that DUSP4 mRNA overexpression promoted proliferation, migration, and tumorigenicity of ccRCC cells [14]. Likewise, Laczmanska et al. compared expression of the DUSP4 gene in RCC and paired healthy tissues and reported DUSP4 gene overexpression in all stages of RCC [15]. The results of these previous studies suggest that DUSP4 acts as an oncogenic factor in RCC. However, the associations of DUSP4 protein expression in RCC tissue with clinicopathological parameters and prognosis have not been reported.

In this study, we performed immunohistochemical (IHC) staining to investigate DUSP4 expression in human RCC tissues. The associations between expression of DUSP4 and clinicopathological characteristics were analyzed in ccRCC. In addition, survival analyses were performed to reveal the prognostic significance of DUSP4 expression.

## 2. Materials and Methods

### 2.1. Patient Selection and Pathological Evaluation

This study included patients who were diagnosed with RCC and underwent curative surgery from June 2006 to March 2017, at Hanyang University Hospital in Seoul, Korea. A total of 252 cases was enrolled retrospectively, and eight cases without follow-up data or paraffin blocks were excluded. The medical records of the remaining 244 patients were reviewed to obtain clinical information, including age, sex, death date, and date of recurrence or metastasis. Two pathologists (Wi Y.C. and Shin, S.-J.) reviewed the tissue slides and pathologic reports. Histological subtypes, histologic grade, tumor size, lymphovascular invasion, tumor necrosis, sarcomatoid features, pT stage, and pN stage were evaluated. We assessed histological grade using the World Health Organization/International Society of Urological Pathology (WHO/ISUP) grading system, and the pathological stage was classified according to the 8th edition of the American Joint Committee on Cancer (AJCC) [16].

### 2.2. Tissue Microarray Construction

We reviewed H&E-stained RCC slides for tissue microarray (TMA) construction. H&E-staining slides were prepared according to the manufacturer’s instructions included in the fully automated system with ready-to-use reagents (Dako CoverStainer, Glostrup, Denmark) at the time of diagnosis of RCC. There were at least four slides containing tumor tissues, and we reviewed all of them for this study. Subsequently, the most representative and non-necrotic areas were selected, and formalin-fixed paraffin-embedded (FFPE) tissue blocks for these slides were collected. Tissue cylinders (3.0 mm in diameter) were collected from each donor block and transferred into the recipient block (Tissue Microarray Set, Labro, Seoul, Korea).

### 2.3. Immunohistochemical Staining and Interpretation

Sections (4 μm thick) were obtained from each TMA block, and we performed deparaffinization and rehydration with routine techniques (e.g., immersing in xylene and graded ethanol). Then, antigen retrieval (heat-induced, 100 °C for 20 min in sodium citrate buffer) and blocking of endogenous peroxidase activity (for 15 min in S2023 peroxidase-blocking solution, Dako, Glostrup, Denmark) were performed. For IHC staining of DUSP4, rabbit anti-DUSP4 polyclonal antibody (1:200, ab72593, Abcam, Cambridge, MA, USA) was used. Detection was achieved using an EnVision Detection System (K5007, Dako, Glostrup, Denmark). A series of IHC staining procedures were performed according to the manufacturer’s instructions using a Bond Max Automated Immunostainer (Leica Biosystems, Nussloch, Germany). DUSP4 expression was defined as nuclear staining of tumor cells. We classified each case as DUSP4 negative (lack of staining or staining in <10%) or DUSP4 positive (staining in ≥10%). There is no standard cutoff for DUSP4 expression in RCC. However, it is common to set a cutoff of 10% for novel biomarkers [17,18]. The staining intensity was not assessed in this study due to concerns of subjectivity and reproducibility. Two pathologists (Bang, S. and Paik, S.S.) evaluated DUSP4 expression without consideration of clinicopathological data.

### 2.4. Online Analysis Based on the Cancer Genome Atlas

We used the Kaplan–Meier plotter (KM plotter, accessed on 28 July 2021, http://kmplot.com/analysis/) to predict the prognostic value of mRNA expression of DUSP4. KM plotter is an online survival analysis tool based on The Cancer Genome Atlas (TCGA) RNA-Seq data. Using this tool, the ccRCC cases included in the database were divided into two cohorts (high or low expression) according to level of mRNA expression. In addition, the clinical outcome of each cohort was evaluated through the survival curve and median survival.

### 2.5. Statistical Analyses

All statistical analyses were performed using SPSS software version 25.0 (IBM, Armonk, NY, USA). The correlations between clinicopathological variables and DUSP4 expression were analyzed using Pearson’s chi-square (χ^2^) and Fisher’s exact tests. The Kaplan-Meier method with log-rank test was performed to evaluate cancer-specific survival (CSS) and recurrence-free survival (RFS). The Cox proportional hazard model was used to determine the prognostic parameters. A two-tailed *p* value less than 0.05 was considered statistically significant.

## 3. Results

### 3.1. The Clinicopathological Characteristics of RCC Patients

Most of the histological subtypes were ccRCC (200 cases, 82.0%). The average age of the patients was 58 years (range: 25–85), and the male to female ratio was 2.17:1. According to the WHO/ISUP grading system, 29 cases (13.0%) were grade 1, 113 (50.7%) were grade 2, 65 (29.1%) were grade 3, and 16 (7.2%) were grade 4. According to the 8th AJCC staging system, the majority of RCC cases was pT1 (75.4%), and there were four cases of RCC (1.6%) with lymph node metastasis. The clinicopathological characteristics of RCC patients are summarized in Table 1.

### 3.2. DUSP4 Expression Pattern According to Histological Subtype

**Figure 1:**
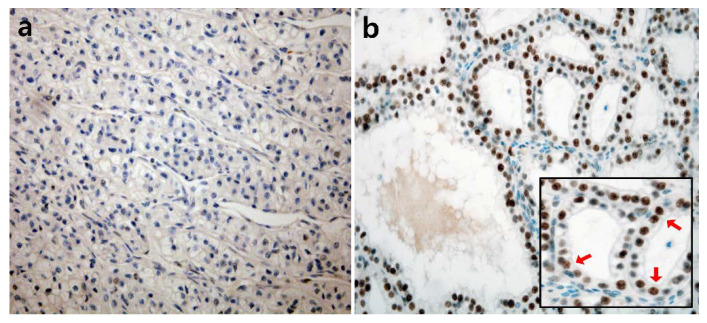
Representative photomicrographs of immunohistochemical staining for DUSP4 in clear cell RCC ((**a**): negative, (**b**): positive, ×400). Nuclei of tumor cells clearly showing brown color were considered as positive staining (indicated by red arrows).

**Figure 2:**
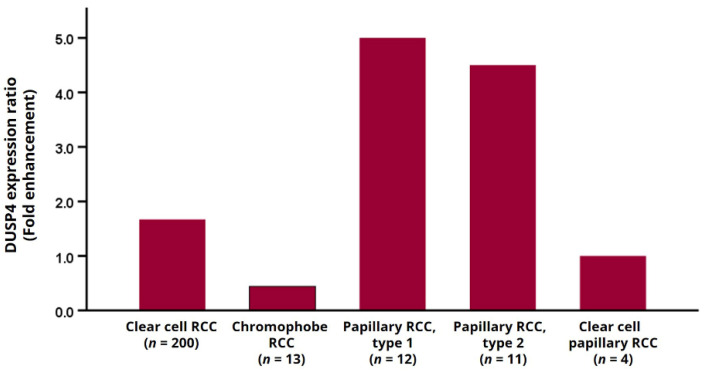
The ratio of DUSP4 positive to DUSP4 negative cases in RCC according to histological subtypes.

The nuclear expression of DUSP4 in RCC was detected by IHC, and representative photomicrographs are presented in Figure 1. Of the total, 154 cases (63.1%) were DUSP4 positive, and 90 cases (36.9%) were DUSP4 negative. Positive cases showed diffuse moderate to strong nuclear staining intensity, but negative cases showed complete negative nuclear staining intensity or weak nuclear staining intensity in some tumor cells (less than 10%). In most histological subtypes, including ccRCC, there were more DUSP4 positive cases than DUSP4 negative cases, but in chromophobe RCC, negative DUSP4 expression was more frequently observed (Figure 2). Positive cases with histological subtypes other than clear cell RCC are presented in Figure S1 and DUSP4 expression according to histologic subtype is summarized in Table 2.

### 3.3. DUSP4 Expression and Clinicopathological Characteristics in ccRCC Patients

The correlations between DUSP4 expression and clinicopathological characteristics in ccRCC are summarized in Table 3. Negative DUSP4 expression was significantly associated with old age (*p* = 0.033), high WHO/ISUP grade (*p* < 0.001), presence of tumor necrosis (*p* < 0.001), and high pT category (*p* < 0.001). Other clinicopathological parameters (sex, sarcomatoid feature, and nodal metastasis) were not associated with DUSP4 expression.

### 3.4. DUSP4 Expression and Clinicopathological Characteristics in ccRCC Patients

**Figure 3:**
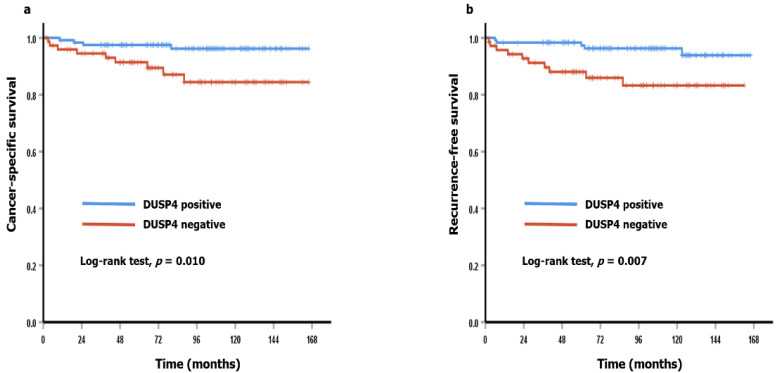
Survival analyses using the Kaplan-Meier method for ccRCC. (**a**) Cancer-specific survival (Log-rank test, *p* = 0.010). (**b**) Recurrence-free survival (Log-rank test, *p* = 0.007).

**Figure 4:**
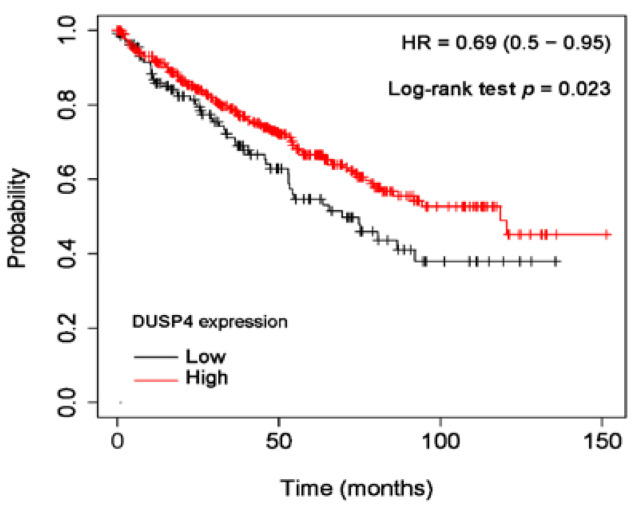
Online analysis with the Kaplan-Meier plotter indicated that low mRNA expression of DUSP4 suggested a poor prognosis in ccRCC patients (*n* = 530; Log-rank test, *p* = 0.023).

Univariate Cox proportional regression analyses were performed to investigate the prognostic significance of DUSP4 expression and clinicopathological parameters in ccRCC cases (Table 4). As a result, short CSS was associated with negative DUSP4 expression (*p* = 0.018), high WHO/ISUP grade (*p* = 0.001), presence of tumor necrosis (*p* < 0.001), presence of sarcomatoid feature (*p* < 0.001), high pT category (*p* < 0.001), and presence of nodal metastasis (*p* < 0.001). In addition, negative DUSP4 expression (*p* = 0.012), high WHO/ISUP grade (*p* = 0.027), presence of tumor necrosis (*p* = 0.001), high pT category (*p* < 0.001), and presence of nodal metastasis (*p* < 0.001) were associated with short RFS. However, negative DUSP4 expression did not act as an independent prognostic factor in multivariate Cox proportional regression analyses (data not shown). In survival analyses using the Kaplan-Meier method, negative DUSP4 cases showed significantly worse CSS and RFS (*p* = 0.010 and *p* = 0.007, respectively) than positive cases (Figure 3). Online analysis with the KM plotter demonstrated that ccRCC patients with low mRNA expression showed significantly shorter overall survival (*n* = 530, *p* = 0.023) (Figure 4).

## 4. Discussion

In our study, we performed IHC staining on human RCC tissues, and each case was assigned to either a DUSP4 positive or negative group. DUSP4 positive cases were more common in most histological subtypes except chromophobe RCC. Negative DUSP4 expression in ccRCC patients was significantly associated with old age, high histologic grade, tumor necrosis, high pT category, and worse clinical outcomes. Our results are contrary to the results of previous studies [14,15]. Similarly, online analysis (KM plotter) using TCGA RNA-Seq data demonstrated that low mRNA expression was associated with a poor prognosis.

DUSP4 is considered a biomarker in various cancer studies, but its precise role is controversial. Previous studies have been performed mainly on breast cancer [19,20,21,22], colorectal cancer [23,24,25,26], lung cancer [27,28], and thyroid papillary carcinoma [12,13,29,30]. Many studies reported that DUSP4 showed an oncogenic effect. However, some studies reported that DUSP4 acted as a tumor suppressor. Gene and protein loss of DUSP4 was found in breast cancer tissues compared to matched normal breast tissues [22]. Downregulation of DUSP4 was noted in the deep region rather than in the superficial region in colorectal cancer [24]. In a study on papillary thyroid carcinoma, high DUSP4 expression suggested a better clinical outcome [30]. In addition, studies on the relationship between DUSP4 expression and drug resistance were performed. Most showed a positive correlation between DUSP4 expression and chemoresistance [21,31,32,33,34,35], and some reported that depletion of DUSP4 reduced the therapeutic effect [36,37].

The DUSP4 gene is located at 8p12; according to data from TCGA’s Pan-Cancer Atlas studies, several genetic alterations have been identified in a small percentage (3.3%) of cancer cases. The most common alteration was deletion of the DUSP4 gene (2.4%), followed by mutation (0.5%) and amplification (0.4%). However, due to their short half-life, the protein level of DUSPs can be affected by post-translational modifications [8]. Promoter methylation of the DUSP4 gene associated with loss of DUSP4 expression has been reported in several types of cancer, including diffuse large B-cell lymphoma [30,38,39].

The effect of DUSP4 expression on carcinogenesis and prognosis in RCC is unclear. In two previous reports, the oncogenic effect of DUSP4 in RCC was considered. They reported that DUSP4 mRNA expression was higher in RCC tissues and cell lines than in normal tissues and tubular epithelial cell lines [14,15]. However, in our study of DUSP4 expression at the protein level, negative expression of DUSP4 was correlated with aggressive clinicopathological features and poor prognosis in human ccRCC. Our results suggest that downregulation of DUPS4 is associated with tumor progression. Our results were confirmed by online survival analysis using TCGA data. The exact molecular mechanisms of DUSP4 downregulation are unknown, although 8p deletion might contribute to DUSP4 downregulation and has been reported with varying frequencies (10–77%) in RCC [40]. Although the association between 8p deletion and expression of DUSP4 in RCC has not been studied, loss of 8p was associated with downregulation of DUSP4 in breast cancer and EGFR-mutant lung adenocarcinoma [22,41,42]. In addition, epigenetic change (promoter hypermethylation) can act as a mechanism to decrease DUSP4 expression.

Dysregulated DUSP4 expression can be involved in various cellular processes by affecting the activity of MARKs. Therefore, for various cancers, DUSP4 is being considered as a therapeutic target or potential biomarker for predicting clinical outcomes. In this study on RCC, loss of DUSP4 expression suggested a poor prognosis, which was evaluated by IHC. This method for detecting DUSP4 is performed with formalin-fixed tumor tissue, and therefore it can be performed simultaneously with or after the diagnosis of RCC. In addition, the results are expressed in the nucleus, making them easily recognized by pathologists and highly reproducible.

In conclusion, we revealed that negative DUSP4 expression is associated with unfavorable clinicopathological characteristics (old age, high WHO/ISUP grade, tumor necrosis, and high pT category) in ccRCC patients. In addition, survival analysis showed that negative DUSP4 expression was associated with poor clinical outcomes. Further studies are needed to determine the exact role and molecular mechanism of DUSP4 in RCC.

## Figures and Tables

**Table 1:** Baseline clinicopathological characteristics of RCC patients.

Characteristics	Case No. (%)
Age, median (range, year)	58 (25–85)
Sex	
Male	167 (68.4%)
Female	77 (31.5%)
Tumor size, mean (range, cm)	3.8 (0.7–15.5)
Histological subtype (by WHO classification, 2016)	
Clear cell RCC	200 (82.0%)
Chromophobe RCC	13 (5.3%)
Papillary RCC, type 1	12 (4.9%)
Papillary RCC, type 2	11 (4.5%)
Others *	8 (3.3%)
Histologic grade (WHO/ISUP grade in clear and papillary RCC)	
Grade 1	29 (13.0%)
Grade 2	113 (50.7%)
Grade 3	65 (29.1%)
Grade 4	16 (7.2%)
Vascular invasion	
Absent	208 (85.2%)
Present	36 (14.8%)
Tumor necrosis	
Absent	205 (84.0%)
Present	39 (16.0%)
Sarcomatoid features	
Absent	228 (93.4%)
Present	16 (6.6%)
pT category	
pT1	184 (75.4%)
pT2	12 (4.9%)
pT3	47 (19.3%)
pT4	1 (0.4%)
pN category	
pN0	240 (98.4%)
pN1	4 (1.6%)

RCC: renal cell carcinoma; WHO, World Health Organization; ISUP, International Society of Urological Pathology. * Other histological subtypes: clear cell papillary renal cell carcinoma, collecting duct carcinoma, acquired cystic disease–associated renal cell carcinoma.

**Table 2:** Differences in DUSP4 expression by histological subtype.

Histological Subtypes	DUSP4 Expression	
	Negative (%) (*n* = 90)	Positive (%) (*n* = 154)
Clear cell RCC	75 (37.5%)	125 (62.5%)
Chromophobe RCC	9 (69.2%)	4 (30.8%)
Papillary RCC, type 1	2 (16.7%)	10 (83.3%)
Papillary RCC, type 2	2 (18.2%)	9 (81.8%)
Clear cell papillary RCC	2 (50%)	2 (50%)
Collecting duct carcinoma	0 (0%)	1 (100%)
Acquired cystic disease-associated RCC	0 (0%)	3 (100%)

RCC: renal cell carcinoma.

**Table 3:** Correlation between DUSP4 expression and clinicopathological characteristics in ccRCC.

Variables	DUSP4 Expression		*p* Value
	Negative (%)	Positive (%)	
Age			0.033
<65 years	43 (32.3%)	90 (67.7%)	
≥65 years	32 (47.8%)	35 (55.2%)	
Sex			0.968
Female	23 (37.7%)	38 (62.3%)	
Male	52 (37.4%)	87 (62.6%)	
WHO/ISUP grade			<0.001
Grade 1 and 2	33 (26.4%)	92 (73.6%)	
Grade 3 and 4	42 (56.0%)	33 (44.0%)	
Tumor necrosis			<0.001
Absent	53 (31.9%)	113 (68.1%)	
Present	22 (64.7%)	12 (35.3%)	
Sarcomatoid feature			0.064
Absent	67 (35.8%)	120 (64.2%)	
Present	8 (61.5%)	5 (38.5%)	
pT category			<0.001
pT1 and pT2	48 (30.6%)	109 (69.4%)	
pT3 and pT4	27 (62.8%)	16 (37.2%)	
pN category			0.684
pN0	74 (37.6%)	123 (62.4%)	
pN1	1 (33.3%)	2 (66.6%)	

**Table 4:** The univariate Cox regression analyses for cancer-specific and recurrence-free survival in ccRCC.

Variables	Cancer-Specific Survival			Recurrence-Free Survival		
	HR	95% CI	*p* Value	HR	95% CI	*p* Value
DUSP4 expression (positive vs. negative)	4.170	1.283–13.559	0.018	3.972	1.355–11.643	0.012
Age group (<65 vs. ≥65)	2.047	0.741–5.655	0.167	2.089	0.756–5.772	0.156
Sex (female vs. male)	1.720	0.485–6.097	0.401	2.829	0.638–12.537	0.171
WHO/ISUP grade (I and II vs. III and IV)	13.423	3.009–59.870	0.001	3.237	1.147–9.138	0.027
Tumor necrosis (absent vs. present)	16.355	5.177–51.667	<0.001	6.136	2.207–17.064	0.001
Sarcomatoid feature (absent vs. present)	20.318	7.316–56.432	<0.001	3.726	0.840–16.519	0.083
pT category (pT1–2 vs. pT3–4)	55.296	7.267–420.728	<0.001	18.732	5.275–66.520	<0.001
pN category (pN0 vs. pN1)	54.804	13.264–226.446	<0.001	95.664	8.674–1055.014	<0.001

## Supplementary Materials

The following is available online at https://www.mdpi.com/article/10.3390/diagnostics11101939/s1, Figure S1: Representative photomicrographs of immunohistochemical staining for DUSP4 in other histological subtypes of RCC.
