# Supplementary material for: Loss of DUSP4 Expression as a Prognostic Biomarker in Clear Cell Renal Cell Carcinoma
Source: Diagnostics (Basel). 2021 Oct 19;11(10):1939. doi: 10.3390/diagnostics11101939 (PMC8534388; doi:10.3390/diagnostics11101939)
Supplement: Supplementary file 1 [file diagnostics-11-01939-s001.zip › diagnostics-1390852-supplementary.pdf]

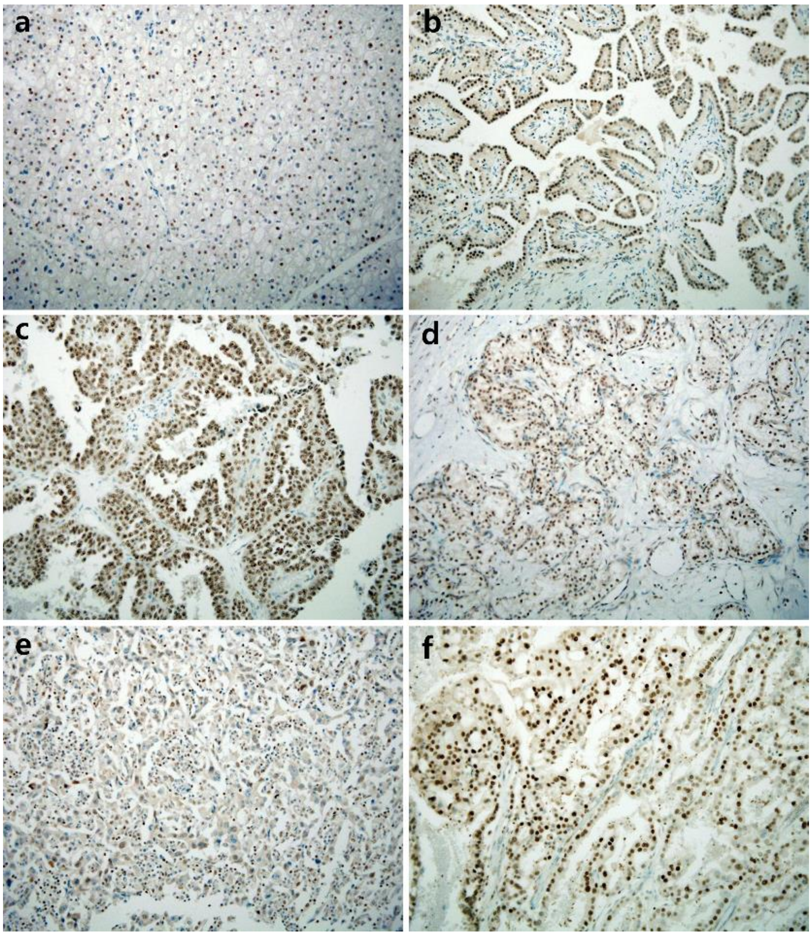

Figure S1. Representative photomicrographs of immunohistochemical staining for DUSP4 in other histological subtypes of RCC ((**a**): Chromophobe RCC, (**b**): Papillary RCC, type 1, (**c**): Papillary RCC, type 2, (**d**): Clear cell papillary RCC, (**e**): Collecting duct carcinoma, (**f**): Acquired cystic disease-associated RCC ×200).
